# Status of Home Delivery and Its Associated Factors among Women Who Gave Birth within the Last 12 Months in East Badawacho District, Hadiya Zone, Southern Ethiopia

**DOI:** 10.1155/2020/4916421

**Published:** 2020-08-19

**Authors:** Deneke Delibo, Melake Damena, Tesfaye Gobena, Bahailu Balcha

**Affiliations:** ^1^Hadiya Zone Health Department, Shone Town Administration Health Office, Ethiopia; ^2^Haramaya University, College of Health Sciences and Medicine, School of Public Health, Harer, Ethiopia; ^3^Wolaita Sodo University, College of Health Sciences and Medicine, School of Public Health, Wolaita Sodo, Ethiopia

## Abstract

**Background:**

Home delivery is responsible to maternal mortality due to obstetric complication like hemorrhage, hypertensive disorders, and sepsis. The prevalence of home delivery is remained very high both nationally (73%) and regionally (SNNPR) with 74.5%. Efforts were made to increase institutional delivery through skilled birth attendance. But women still prefer home as a place of delivery. This study was done to determine whether home preference has association with home delivery or not and the reason why they prefer home delivery

**Method:**

A community-based cross-sectional study was conducted in East Badawacho District from January 26 to February 25/2018. A total of 552 participants were selected by systematic sampling. Data were collected using both quantitative and qualitative methods. Bivariate and multivariable analyses were carried out to identify factors associated with home delivery. Qualitative data was analyzed thematically, and results were triangulated with the data. Associations were determined by using OR at 95% CI and *p* value at 0.05.

**Result:**

Home delivery is found to be 73.6% (95% CI, 69.9%-77.2%). Lack of written birth plan for birth preparedness and readiness (AOR = 14.965, 95% CI: 4.488-49.899), incomplete number of ANC visits (1-3)(AOR = 4.455, 95% CI: 1.942-10.221), and home preference as a place of delivery (AOR = 4.039, 95% CI: 1.545-10.558) were independent predictors of home delivery.

**Conclusion:**

Home delivery was high in the district. The independent factors significantly associated with home were lack of written birth plan for preparedness and readiness, incomplete number of ANC visits (1-3), and home preference as place of delivery. Actions targeting maternal education, encouraging number of ANC visits, and avoiding barriers for ID utilization were the crucial areas to tackle the problem.

## 1. Introduction

Home delivery is a practice of childbirth in a nonclinical setting that takes place in a residence [1]. It plays a vital role as the place to maternal death occurrence due to obstetric complication like hemorrhage, hypertensive disorders, sepsis, abortion, embolism, and all other direct causes of death [2].

Health conditions related to pregnancy and childbirth, combined with HIV/AIDS, are the leading causes of death among women in developing regions [3]. An estimated global total of 10.7 million women have died due to maternal causes. Developing regions account for approximately 99% of the estimated global maternal deaths in 2015, with sub-Saharan Africa alone accounting for roughly 66%, followed by Southern Asia [4].

The main adverse outcomes in patients admitted due to obstetric complication after home delivery were PPH in 48% patients (of which primary 61.2% and secondary in 38.8% patients) followed by retained placenta/placental tissues in 26% of women. Three women died out of the total of 261 admitted patients (1.1%) due to puerperal sepsis within a few hours of admission which is a very high frequency of maternal deaths among patients who delivered at home [2].

About 73% of all maternal deaths were due to direct obstetric causes like hemorrhage, hypertensive disorders, sepsis, and abortion, but the top three leading causes are hemorrhage, hypertensive disorders, and sepsis which are responsible for more than 50% of maternal deaths worldwide [5].

Factors that prevent women from receiving adequate health care during pregnancy and childbirth include limited availability and poor quality of health services, a lack of information on available services, certain cultural beliefs and attitudes, and poverty [4].

The study conducted in Pakistan revealed almost 60% of women who gave birth at home had received no antenatal care while 37.5% had received infrequent/irregular check-ups by qualified medical personnel. The most frequent quoted reasons for home delivery were family tradition (72.8%) and lack of affordability (68.6%) [2].

Maternal health has improved considerably over the years, yet in Southern Asia and sub-Saharan Africa, only half of pregnant women have adequate care during childbirth [3]. The number of women dying due to complication during pregnancy and childbirth decreased by nearly 50% in 2013 while such progress is notable, the average annual rate of decline is far below needed to achieve millennium development goal targets (5.5%), and the number of deaths remains unacceptably high [6].

The magnitude of home delivery varies from place to place. The status of home delivery in Pakistan was 74.3% [7] and 62% in Kenya. It was almost similar nationally (73%) and regionally (SNNPR, 74.5%). The SNNPR was the 4th highest with home delivery status at national level following Afar region (85%), Somali region (82%), and Oromia region (81%) [8].

Home delivery is responsible for high numbers of maternal and newborn deaths by being the place of occurrence of obstetric complications during labor and delivery with the help of traditional birth attendants [9].

About 830 women are dying every single day due to the complications of pregnancy and childbirth. The estimated average global MMR was 216 per 100,000 births, and sub-Saharan estimated average MMR was 546 per 100,000 births. The World Health Organization (WHO) reported that Ethiopia has one of the highest maternal and neonatal mortality rates in the world [4]. The point estimated pregnancy-related mortality was 412 deaths per 100,000 live births during seven years before the survey. This indicates that for every 1000 live births, approximately four women died during pregnancy and child birth and within 42 days after termination of birth. Maternal death rate in Ethiopia shares 25% of all female death [8].

Many researches considered home preference as the outcome variable, but no study was done to identify home preference whether it is associated with home delivery or not. This study tried to analyze whether home preference was a predictor for home delivery or not and the reasons behind to prefer home to give birth in rural district of East Badawacho.

## 2. Methods and Materials

The community-based cross-sectional study was employed in East Badawacho, which is one of rural districts in Hadiya Zone, Southern Ethiopia, located at 342 km and 277 km in the south of Addis Ababa through Shashamane and Butajira, respectively, about 121 km far from Hawassa and situated at 97 km East from the Zonal capital of Hosanna. The study used both quantitative and qualitative methods from Jan 26 to Feb 25/2018.

Sample size was determined by using a single population proportion formula at a 95% confidence level, 80% power of the test, and 5% margin of error, where *p* is the proportion of population who gave birth at home (*p* = 79.4%), from a similar study in Arbaminch Zuria District [10], and by considering 2 for the design effect and 10% for nonresponse rate (the final sample size (*n*) = 552).

There are 35 kebeles in the east Badawacho District. First, 12 kebeles were selected by using simple random sampling technique (Lottery method). The total number of women who gave birth in the selected kebeles from January 2017 up to January 2018 was identified by using HP family folder registration and immunization registration book as a sampling frame. The sample size in each kebele was allocated proportionally to the total number of deliveries in each kebele for the selected kebeles. Then, study participants were selected by using a systematic sampling method through a respective kebele's 3^rd^ respondent in each kebele and interviewed by diploma level nurses using a structured questionnaire adapted from different literatures.

The dependent variable was home delivery while the following factors were included in the model as independent variables: sociodemographic factors (age, occupation, marital status, wealth status, and level of education), maternal and obstetric characteristics (ANC visits, parity and previous birth at home and preferred place to give birth), knowledge and attitude factors (maternal awareness, attitude, decision-making power, birth plan, and media exposure), and health facility-related factor (privacy and confidentiality, travel time/distance, counseling service/approaching behavior of staff, and waiting time to get service).

The questionnaire was prepared in English, translated to local Hadiyya language to make it understandable by the study participants and then was retranslated to English by another person to check whether the translation was consistent. Data collectors were supervised by supervisors; 5% of the questionnaire was pretested at Sibeya kebele in West Badawacho District which is not part of the study area.

To supplement the quantitative study, we conducted 5 FGD among 47 participants selected by using purposive sampling technique.

10 participants per FGD were conducted by the investigator himself at the health center:

- 10 participants were selected from women who gave birth within 12 months prior to the survey from age < 35 years
- 10 participants from husbands from five kebeles of the catchment area
- 10 participants from TBAs from five kebeles of the catchment area
- Seven participants from midwives one from each health center's delivery team

First, data were checked manually for its completeness and consistency. Each completed questionnaire was assigned a unique code and entered to EpiData version 3.1. Then, data were exported into SPSS version 20 for data processing and analysis. Frequencies, proportions, and summary statistics were used to describe the study population in relation to relevant variables and presented in tables. Bivariate analysis was carried out to identify variables that are significantly associated with home delivery. Those variables in bivariate analysis at *p* value < 0.25 were included in multivariate logistic regression; then, multivariate logistic regression analysis was performed for those factors that showed a statistically significant association in bivariate analysis. Finally, variables whose *p* < 0.05 in logistic regression were considered to have statistically significant association. Data was analyzed using thematic manual analysis.

The study was approved by College of Health and Medical Sciences, Institutional Health Research Ethics Review Committee (IHRERC), Haramaya University. The supportive letter was written from the college to SNNPR health bureau. Participants were informed clearly about the purpose, risk, and benefit of the study, and informed, voluntary, written, and signed consent was obtained from them. The confidentiality of responses was maintained throughout the research process by giving a code for each participant. Personal privacy and cultural norms were respected.

## 3. Result

A total of 531 women who gave birth within the last 12 months were involved in this study that makes a response rate of 96.2%. The mean (±SD) age of study participants was 26.88 (±4.506), and about 60% of mothers were ≥25 years old. Almost all respondents were married (522 (98.3%)), and the rest were widowed (9 (1.7%)). Moreover, 233 (43.9%) and 167 (31.5%) of respondents and their husbands had never attended formal education, respectively. Majority of the respondents (62.1%) and their husbands (86.6%) were housewives and farmers, respectively. Wealth quintile was determined through principal component analysis (PCA). The 1^st^ (poorest) and 5^th^ (richest) quintiles had the same percentage (20%) as shown in Table 1.

### 3.1. Maternal and Obstetric Characteristics of the Respondents

Among 531 respondents, 60 (11.3%) of the respondents had single child whereas 186 (35%), 168 (31.7%), and 117 (22%) of the respondents had 2-3, 4-5, and ≥6 numbers of children, respectively. About 81% of respondents attended ANC in their last pregnancy. Among mothers who attended antenatal care, only 147 (34.2%) women completed the minimum requirement number of ANC visits recommended by the WHO. Even though 300 (56.5%) women preferred a health facility to give birth, only 140 (26.4%) mothers gave recent birth at a health facility. The previous place of birth (prior to recent delivery) was home for 395 (83.9%) of respondents among mothers who had two or more children (Table 2).

### 3.2. Knowledge, Attitude, and Practice of Respondents

Majority of women (436 (82.1%)) received health education on maternal health from different sources (HEWs, HWs, radio, etc.). About 70% of the respondents among ANC attendants were informed on danger signs during pregnancy and delivery at a time of their ANC visits. About 369 (85.6%) of the respondents among ANC attendants had trust and confidence on health workers at ANC services given during their ANC follow-up. Majority of respondents (327 (61.6%)) were not exposed at all to mass media per week while respondents exposed at least once and less than once per week were 92 (17.3%) and 112 (21.2%), respectively (Table 3).

### 3.3. Health Facility-Related Factors

The traveling time needed to reach nearby health facility for 505 (95.1%) of the respondents was ≤60 minutes (an hour). Moreover, 111 (21%) of the respondents denied friendly approaching behavior of staff. From all respondents, only 111 (17.3%) were satisfied whereas 255 (61.6%) were unsatisfied with the time spent in the waiting area of the nearby health facility (Table 4).

The result of quantitative part of this study showed that about 15.3% of respondents denied privacy and confidentiality in health facility. This was also raised many times in focused group discussion as the reason for home preference. A 38-year-old woman stated like this: “…privacy is in danger since many professionals encircled the mothers during delivery. The room of service provision is not safe for privacy and does not give comfort for laboring mother. This makes us to feel fear and shame to be exposed (nakedness).why this could be so? That is why the women preferred home delivery.” [woman from Bantewosen kebele].

### 3.4. Magnitude of Home Delivery

This study revealed that 73.6% (95% CI, 69.9%-77.2%) of women gave birth at home their last delivery.

The ambulance-related problems were late arrival or not coming at all, network problem during call, and road difficulty for ambulance. This enforces women to give birth at home as one woman indicated like this: “when call made for ambulance, it doesn't reach on time or not come at all and during night labor, mobile network sometimes may not work. Ambulance driver is not committed to come at night. All these lead to decision of home delivery.”[25-year-old woman from the 1^st^ Amburse kebele].

### 3.5. Multivariate Logistic Regression Analysis

The factors significantly associated with home delivery at their *p* value < 0.05 were written birth plan on birth preparedness and complication readiness, numbers of ANC visits, and preferred place to give birth (home preference). As you can see from the Table 5, mothers who did not have written birth plan on birth preparedness and complication readiness were about 15 times more likely to practice home delivery than mothers who had written birth plan on birth preparedness and complication readiness (AOR = 14.965, 95% CI: 4.488-49.899). Respondents with incomplete ANC visits(1-3) were about 4.5 times more likely to give birth at home than mothers with complete ANC visits (≥4) (AOR = 4.455, 95% CI: 1.942-10.221). Mothers with home preference were 4 times more likely to give birth at home as compared to those mothers who preferred health facility as the place of their delivery (AOR = 4.039, 95% CI: 1.545-10.558). (Table 5).

### 3.6. Reasons for Home Delivery Preference from Qualitative Result

A total of 40 participants 8, 7, 9, 9, and 7 involved husbands, TBAs, women aged <35 years, women aged ≥35 years, and midwives or health workers (delivery ward), respectively. The participants were in an age range of 20–75 years old. Out of 40 interviewees, 29 (72.5%) of them were female while the rest (11 (27.5%)) were male from FGDs. Interviewees' education status was no formal education, primary [1–8], secondary [9–12], and college and above for 8 (20%), 14 (35%), 5 (12.5%), and 13 (32.5%) of the respondents, respectively. The average time taken for discussions among all group discussions was 52 minutes.

The issue of health professional's skill and their negligence with verbal abuse (poor interaction of providers and clients) were mentioned as the reasons to home delivery preference. A 36-year-old mother stated that: “ I visited health facility with chief complaint of pain of labor. After examined my abdomen, health worker had said that you are still in 7th week of gestation so that you will come back 2 weeks later. When I refused to do so and asked for service, he started to insult me and then I left him alone in the room to go my home. While I was on the way of my home, the pain became severe and then, I went to TBA. She said that the labor is started so, go to your home; I will come soon to you. About 30 minutes later, I gave birth in my home with her attendance. This is due to unskilled health worker which made me to compare uneducated TBA with health workers. So, TBA is preferred in my opinion.” [mother from A/Anjulo kebele]. This consolidates the quantitative study in which about 21% of respondents denied the friendly approaching behavior of the staff.

Another most important reason was TBA preference as the birth attendant during delivery. This is because of TBA's good friendly approaching behavior and interaction to comfort laboring women. A 23-year-old mother from FGD participants stated that “when we feel pain of labor, TBA comes to our home and be with us until we give birth. They also try to relief pain by massaging with butter and give us comfort which help us to ignore pain. Being with own's own family is another benefit of giving birth at home. No cold attack, fear of shame and issue of privacy. TBAs don't expose us naked but cover with cloth. However, this is not accustomed by health workers in health facility so that our preference is home to be attended by TBAs” [from Lakole kebele of the <35-aged group].

## 4. Discussion

The results of this study showed that home delivery was very high in the study population. This result was consistent with other studies conducted in Pakistan (74.3%) [7], EDHS 2016 report (73.3%) [8], and Gozamin District in Amhara region (75.3%) [11], but lower than a study conducted in Arbaminch Zuria District (79.4%) [10], zone 3 of Afar region (83.3%) [12] and higher than the result from, Zala woreda, Southern Ethiopia (67.6%) [13], Anlemo District, Hadiya Zone (49.3%) [1], and Wolaita and Dawro Zone (62%) [14]. This difference could partly be explained by their differences in socioeconomic, geographical location and the time gap between the studies of the stud populations.

Home delivery was significantly influenced by lack of written birth plan on birth preparedness and complication readiness. Mothers who had not written a birth plan were more likely to give birth at home than their counterparts. This may be due to the fact that preparing one's own self by written plan may enhance recall to the expected date of delivery as well as readiness to respond any problem during labor thereby reduce home delivery. This finding is similar with a study conducted in Gozamin District, Northwest Ethiopia [11].

This study also revealed that the number of incomplete ANC visits was another predictor of home delivery. Those mothers with an incomplete number of ANC visit (≤3 visits) were more likely to give birth at home than mothers who had completed (≥4) their ANC visit. The reason may be due to the fact that the counseling about written birth plans where to give is component of and provided at last ANC visit according to WHO recommendation. Therefore, those mothers with incomplete visits are probably prone to practice home delivery. This finding is also in line with different studies in Afghanistan, Ethiopia, and some African countries [13, 15].

Home preference to give birth was found to be a predictor of home delivery in this study. Mothers who preferred home as the place of their delivery were more likely to give birth at home than mothers who preferred a health facility. This is due to different subjective reasons of respondents in their natural setting.

A mother could give birth at home even though her preference is a health facility in some conditions like the nature of labor (sudden labor, rapid onset of labor, and night labor) whereas she could also give birth at a health facility although her preference is home due to obstetric complications. In the situation where all of the above conditions are kept constant, mothers prefer the place of delivery according to their respective reasons.

Generally, the discussion was intended to explore the participants' reason behind to give birth at home. The raised reasons were the most important and call for health sector stakeholders to shift their strategy to address these obstacles thereby to tackle home delivery preference. This needs multicollaboration with all health service provision structure, political, and social organization.

Our study used both quantitative and qualitative data collection method that widens the scope of the study and one method that counterbalances the shortcomings of the other method. Besides, the selection bias is minimized as a community-based study, and probability sampling was applied. All these could increase the accuracy and contribute greater confidence in the generalizability of findings. Although special attention was paid to retrieve the lists of recently delivered women from family folder and immunization registration book from health post, there is a possibility that some women might have been missed out, especially those women who were nonusers of immunization and from HH with nonupdated family folder prior to the study. Besides, the cross-sectional nature of the study does not allow establishing causality of associations and the results should be interpreted cautiously. Recall bias (even though time interval was minimized to 12 months) and social desirability bias cannot be ruled, which may also be a problem.

## 5. Conclusion and Recommendations

This study revealed that home delivery in last 12 months was high. Lack of written birth plan on birth preparedness and complication readiness, incomplete number of ANC visits (≤3), and home preference of delivery were the predictors for home delivery among the study participants.

The most mentioned reasons for women's preference of home as the place of delivery were ambulance-related problems (late arrival, not coming, and network problem), lack of awareness and knowledge of mothers (fear of shame and operation, nonvisit of ANC, and low maternal health care seeking), health provider-related problem (verbal abuse, lack of skill, late referral, negligence, and poor hospitality), health facility-related problem (long waiting time and lack of privacy and confidentiality), and TBA preference.

In general, the health bureau, zonal health department, and district health office should work as per national plan to increase the institutional delivery by creating a sense of accountability and responsibility in health workers and awareness of pregnant women on the use of institutional delivery.

## Figures and Tables

**Table 1:** Socioeconomic characteristics of respondents who gave birth within the last 12 months in East Badawacho District, Hadiya Zone, Southern Ethiopia 2018 (*n* = 531).

Variable	Categories	Frequency	Percentage
Parity (*n* = 531)	1	60	11.3
	2-3	186	35.0
	4-5	168	31.7
	≥6	117	22.0

ANC attendance (*n* = 531)	No	100	18.8
	Yes	431	81.2

Number of ANC visits (*n* = 431)	Incomplete (1–3)	284	69.6
	Complete (≥4)	147	34.2

Advise on written birth plan for birth preparedness during ANC (*n* = 431)	No	229	53.1
	Yes	202	46.9

Written birth plan for birth preparedness (*n* = 202)	No	171	85.1
	Yes	31	14.9

Your preferred place to give birth for last delivery (*n* = 531)	Home	231	43.5
	Health facility	300	56.5

Place of previous delivery (*n* = 471)	Home	395	83.9
	Health facility	76	16.1

**Table 2:** Maternal and obstetric characteristics of respondents who gave birth within the last 12 months in East Badawacho District, Hadiya Zone, Southern Ethiopia, 2018.

Variable	Categories	Frequency	Percentage
Parity (*n* = 531)	1	60	11.3
	2-3	186	35.0
	4-5	168	31.7
	≥6	117	22.0

ANC attendance (*n* = 531)	No	100	18.8
	Yes	431	81.2

Number of ANC visits (*n* = 431)	Incomplete (1–3)	284	69.6
	Complete(≥4)	147	34.2

Advise on written birth plan for birth preparedness during ANC (*n* = 431)	No	229	53.1
	Yes	202	46.9

Written birth plan for birth preparedness (*n* = 202)	No	171	85.1
	Yes	31	14.9

Your preferred place to give birth for last delivery (*n* = 531)	Home	231	43.5
	Health facility	300	56.5

Place of previous delivery (*n* = 471)	Home	395	83.9
	Health facility	76	16.1

**Table 3:** Knowledge, attitude, and practice of respondents who gave birth within the last 12 months in East Badawacho District, Hadiya Zone, Southern Ethiopia, 2018.

Variable	Categories	Frequency	Percentage
Received health education on maternal health (*n* = 531)	No	95	17.9
	Yes	436	82.1

Informed danger sign of pregnancy during ANC visits (*n* = 431)	No	131	30.4
	Yes	300	69.6

Confidence and trust on health worker during ANC (*n* = 431)	No	62	14.4
	Yes	369	85.6

Giving birth at health facility is preferable to home (*n* = 531)	Negative attitude	388	73. 1
	Neutral	36	6.8
	Positive attitude	107	20.1

Decision maker on place of delivery (*n* = 531)	Me myself	327	61.6
	Husband	99	18.6
	Me and my husband	78	14.7
	Family	27	5.1

Exposure to mass media (*n* = 531)	At least once/week	92	17.3
	Less than once/week	112	21.1
	Not at all	327	61.6

**Table 4:** Health facility-related characteristics of respondents who gave birth within the last 12 months in East Badawacho District, Hadiya Zone, Southern Ethiopia, 2018 (*n* = 531).

Variable	Categories	Frequency	Percentage
Time needed to travel to nearby health facility in minutes	≤60′	505	95.1
	>60′	26	4.9

Friendly approaching behavior of the staff	Poor	111	20.9
	Neutral	48	9.0
	Good	372	70.1

Time spent in waiting area to get service	Satisfied (≤15′)	111	17.3
	Fair (16-30′)	165	21.1
	Unsatisfied (>30′)	255	61.6

Privacy and confidentiality in nearby health facility (*n* = 531)	Poor	81	15.3
	Neutral	35	6.6
	Good	415	78.1

Place of last/recent delivery (*n* = 531)	Home	391	73.6
	Health facility	140	26.4

**Table 5:** Factors significantly associated with home delivery among study participants, from multivariate logistic regression analysis, in East Badawacho District, Hadiya Zone, Southern Ethiopia, 2018.

Variable	Category	Recent delivery place		COR (95% CI)	AOR (95% CI)
		Home (#)	HF (#)		
Awareness on at least one danger signs of pregnancy	No	109	22	2.296 (1.367, 3.857)	1.921 (0.711, 5.191)
	Yes	205	95	*1*	*1*

Number of ANC visits	Incomplete(1-3)	248	36	8.455 (5.246, 13.626)	4.455 (1.942, 10.221)^∗^
	Complete (≥4)	66	81	1	*1*

Written birth plan	No	135	36	10.781 (4.452, 26.110)	14.965 (4.488, 49.899)^∗^
	Yes	8	23	1	1

Advise on written birth plan for birth preparedness during ANC visits	No	177	52	1.689 (1.103, 2.586)	1.537 (0.642, 3.681)
	Yes	135	67	1	1

Time spent in waiting area to get ANC service	Satisfied	79	56	1	1
	Fair	110	30	2.599 (1.531, 4.413)	1.969 (0.643, 6.031)
	Unsatisfied	202	54	2.652 (1.682, 4.181)	2.708 (0.974, 7.530)

Preferred place for the last delivery	Home	203	28	4.319 (2.729, 6.836)	4.039 (1.545, 10.558)^∗^
	Institution	188	112	1	1

Received health education on maternal health	No	76	19	1.537 (0.891, 2.649)	1.283 (768, 2.144)
	Yes	315	121	1	1

^∗^Significant at *p* < 0.05; COR = crude odds ratio; AOR = adjusted odds ratio.

## Data Availability

The data used to support the findings of this study are available from the corresponding author upon request.
